# Model Investigation of Argon Injection into Liquid Steel at Ladle Furnace Station with Using of Innovative Module

**DOI:** 10.3390/ma16247698

**Published:** 2023-12-18

**Authors:** Tomasz Merder, Piotr Warzecha, Jacek Pieprzyca, Marek Warzecha, Robert Wende, Artur Hutny

**Affiliations:** 1 Faculty of Materials Engineering, Silesian University of Technology, Krasinskiego 8, 40-019 Katowice, Poland; tomasz.merder@polsl.pl (T.M.);; 2 Faculty of Production Engineering and Materials Technology, Czestochowa University of Technology, al. Armii Krajowej 19, 42-201 Czestochowa, Poland; piotr.warzecha@gmail.com (P.W.);; 3 Cognor SA Ferrostal Łabędy Gliwice, Anny Jagiellonki Street 47, 44-109 Gliwice, Poland; rwende@ferrostal.com.pl

**Keywords:** ladle furnace, ladle, numerical modeling, physical modeling, numerical procedures

## Abstract

High-quality steels are defined primarily by a small quantity of non-metallic inclusions and a high degree of chemical homogenisation. The ladle furnace (LF) is the most important metallurgical unit in which the quantity of non-metallic inclusions can be significantly reduced while ensuring metal chemical homogenisation. It is achieved largely due to appropriate controlling and the use of increasingly developed inert gas purging techniques. Various types of porous plugs (channel or radial type) are used in the metallurgical ladles. In aggregate units of intermediate-ladle type, various types of channel plugs and/or gas curtains are successfully used. In the research presented herein, a new and innovative module for inert gas injection into liquid steel for deep refining was tested. The presented research relates to the innovative module using to replace the standard porous plug in the steelmaking ladle on the outside-furnace (LF) processing station. Hybrid modelling methods (numerical and physical modelling) were used to carry out research. Module using causes significantly faster alloy additive dispersion in ladle volume compared with the standard solution (the standard porous plug). Furthermore, the obtained flowing structure positively affects liquid steel refining and mixing processes after alloy additive addition. A new technological solution, i.e., gas-injection module—differs from the traditional porous plugs currently used in the steel mills in terms of geometric parameters, external and internal structure, and what is most importantly, terms of the active surface area—shall be understood in as the surface area wherein slots occur.

## 1. Introduction

As civilisation develops, demand for steels with very high metallurgical purity is increasing. High-quality steels are defined primarily by small quantities of non-metallic inclusions and chemical homogenisation of high degree and increasing strength parameters [1,2,3]. The ladle furnace, one of the steelmaking process line components, is the most important metallurgical aggregate unit in which a certain quantity of non-metallic inclusions can be significantly reduced, and simultaneously, satisfactory chemical and thermal homogenisation can be achieved. This is achieved to a large extent through appropriate controlling and using increasingly developed inert gas injection techniques. Various types of porous plugs (channel or radial types) are used in the steelmaking ladles [4,5]. In the research presented herein, a new and innovative module for inert gas injection into liquid steel for deep refining was tested. Striving towards increasing steel refining efficiency necessitates the continuous improvement of our expertise and knowledge about phenomena occurring at various steelmaking technological stages [6,7,8]. The basic and commonly used operation on the LF station is inert gas injecting. The aim is to create the required hydrodynamic conditions promoting chemical composition and liquid steel temperature homogenisation and enhancing the efficiency of the non-metallic inclusion refining process [9,10,11,12].

In industrial conditions, it is difficult to measure most process parameters. Physical modelling and numerical simulation are effective research tools used for the identification of phenomena affecting the course mechanism thereof.

From a physics point of view, the inert gas injection process into liquid steel is complex and requires the identification of several phenomena that must be precisely described in the form of two-phase (liquid-gas) mathematical models. Research efficiency and result quality depend on the quality of these models. Frequently, because of this complexity, previous studies have identified its various stages separately; for example, gas bubble forming manner on porous material surface or slot and mutual interaction thereof, the way bubbles flow towards free steel surface and growth thereof, and gas column impact on hydrodynamic flow conditions in ladle volume. The latter is associated also with gas flow rate determination and the tendency for non-metallic inclusions captured by the bubbles. Another research issue is the way gas cone is formed in liquid steel volume and the dispersion degree thereof. This, in turns, leads to problems associated with metal mirror exposure (open eye) [13]. The phenomena presented directly impact liquid steel purging with the inert gas process, and the nature and course thereof to a large extent depend on the design of the gas-permeable plugs used. Therefore, requirements formulated towards them relate to parameters such as:

- ability to generate the required gas column structure in the liquid steel ensuring effective mixing thereof with possibly limited impact on the steel mirror and the metal-slag interface,
- ability to create large quantities of the possibly fine gas bubbles and, consequently, create gas dispersion with a high degree in liquid steel volume,
- striving to reduce inert gas consumption while ensuring process efficiency.

In this regard, for construction of the modern gas-permeable plugs, extremely sophisticated solutions from both technological and material points of view are used. Therefore, further increase in functionality is limited. The solution to this problem may lie in a new approach to the construction of the gas-permeable plugs and the functioning thereof in the steelmaking ladle space. One drawback of the current solutions is the small active surface area of the gas-permeable plugs. This is due to construction nature thereof. Increasing this surface area could effectively improve plug functionality, however requires an innovative approach to construction thereof.

Nevertheless, the research presented in this paper concerned the use of innovative module replacing traditional porous plugs in the steelmaking ladle in the outside furnace processing station. The present paper is the first from a planned series relating to an innovative method for inert gas purging into liquid steel. To carry out this research, a hybrid modelling method was used. In this method, physical modelling and numerical simulations as elements of a coherent research programme were utilized. Both methods were mutually complementing stages and conducted in interaction. Before proceeding, several preparatory works to effectively utilize the advantages of both modelling methods and achieve greater result reliability were conducted.

## 2. Research Object and Research Methodology

**Figure 1:**
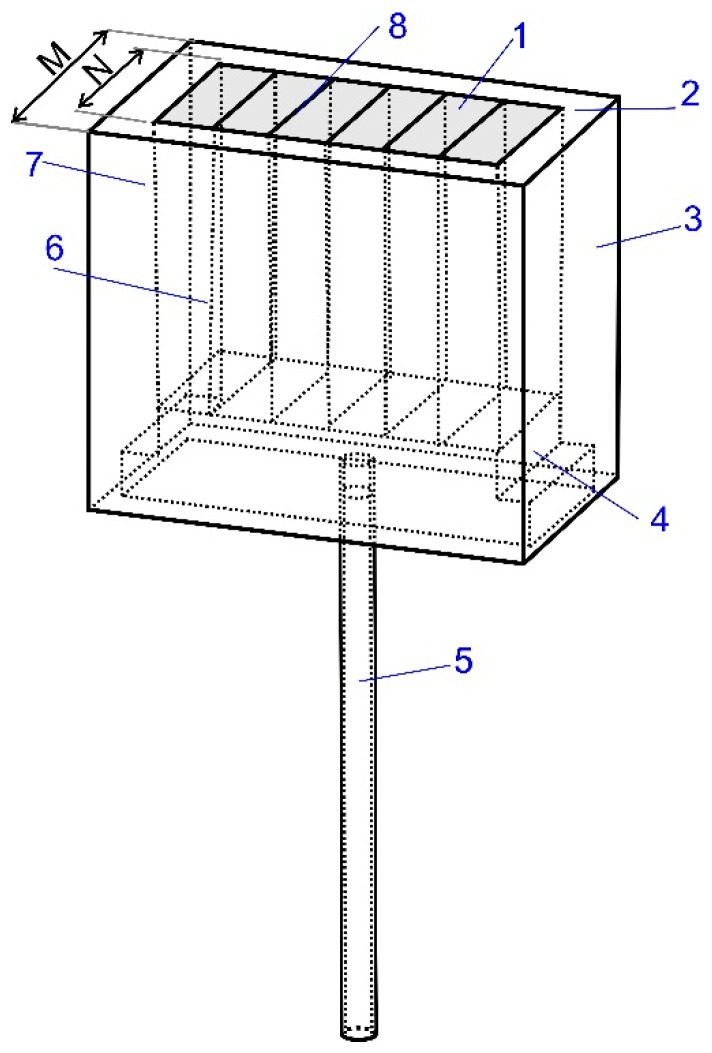
Construction schematic diagram of innovative module for inert gas injection. 1—capillary module, 2—slots, 3—concrete module casing, 4—collector system, 5—argon supply stub, 6—perforation, 7—protection plate and 8—slotted capillaries—slots. Reprinted/adapted with permission from Ref. [14] 2018, Barchuk Y., Shcherbak M.

This study aimed to demonstrate the advantages of using the innovative module for gas purging into the ladles, made according to patent no. PL 229475 [14]. A schematic module diagram is presented in Figure 1. An innovative system for inert gas purging into the melt consists of the capillary module arranged in a manifold to which the gas supply port is connected. Slotted capillaries of determined cross-sections are arranged in the capillary module. The width of the slotted capillaries should not exceed 350 μm. The gaps should be 100 to 300 μm wide. The number of cracks was 8. The ratio of the depth M to N was 0.478.

Figure 1 illustrates the idea of the module construction. It differs from the traditional “slotted” type fitting with an innovative design, a rectangular cross-section. It is characterized by a much larger active surface. Active surface (see Figure 1—gray color) means the area of distribution of crevices on the face of the purging plug (innovative module). The module’s internal structure also differs. It is devoid of any type of porous material and the gas is introduced into the capillary system through a specially constructed collector system, which is a kind of pressure shock absorber between the gas dosing system and the liquid steel. This structure causes a different mechanism of gas column formation. The fragmentation of bubbles is much greater than in previous solutions. The degree of gas dispersion in the model liquid is also much better. It can therefore be concluded that the efficiency of the process is improved compared with existing techniques, and the negative impact of the gas column on the refractory lining and the growth of the slag eye is limited.

Additionally, unlike the traditional porous plug, it does not require the use of a shell fitting, rather, it is directly installed in the counter lining. It is assumed that it will work properly throughout the entire ladle campaign period. The internal structure of the module also ensures proper patency of the slotted capillaries.

Importantly, from the point of view of metallurgical practice, the use of the module ensures the required safety because it is an integral part of the lining.

**Figure 2:**
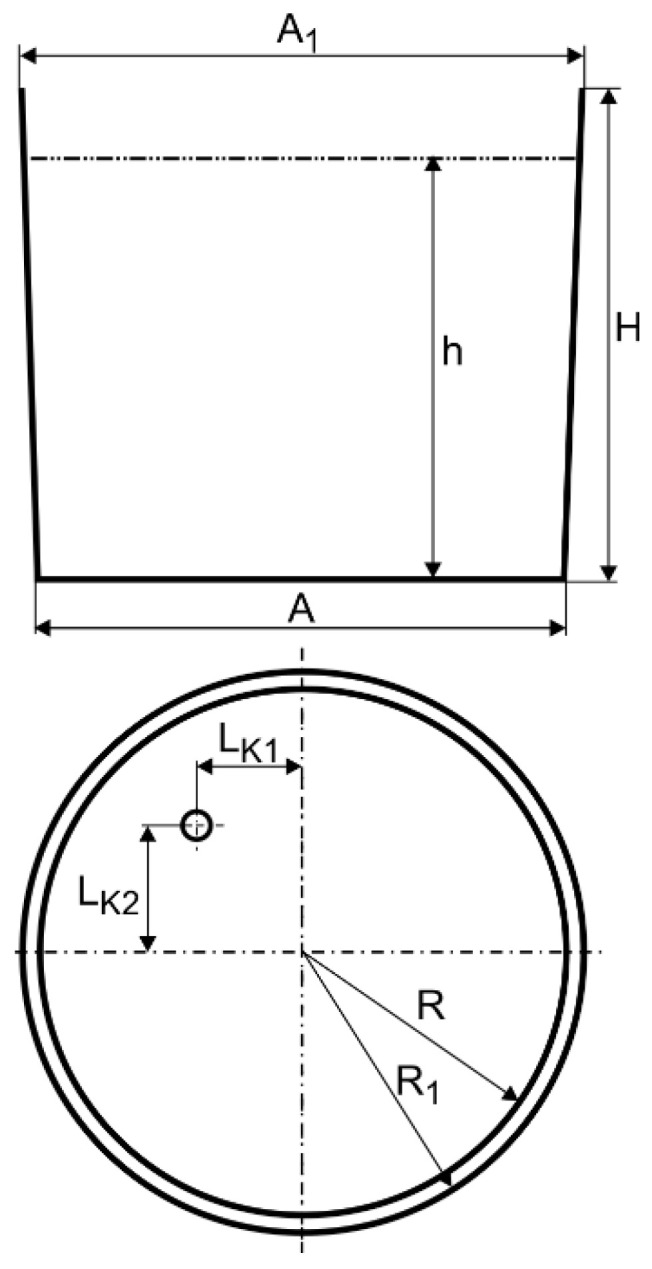
Ladle schematic diagram.

For industrial measurements, the steelmaking ladle with a nominal capacity of 65 tons of liquid steel was used. This ladle furnace station operates in one of the domestic (Polish) steel plants. Essential ladle construction features are shown in Figure 2 and data are given in Table 1. It includes porous plug location (slot type, number of slots—12 on 70 mm circumference, and the innovative module). During the measurement proceeding, process parameters such as average Argon flow rate, metal bath temperature, and average alloy additive quantity were determined.

Based on industrial measurements carried out at ladle furnace station during normal production conditions for steel grades being the research subject, the average gas flow rate (Argon) is 200 dm^3^·min^−1^, the average liquid steel temperature is 1860 K, and the average alloy addition is 100 kg, therefore these values were adopted for farther research.

Industrial process model creating, although relates to the most realistic phenomena, is abstractive by nature. Understanding phenomena occurring in industrial processes and describing thereof in such a manner to obtain desired results requires considerable effort. When creating a process model, in this case mathematical or physical model of inert gas purging into liquid steel, a series of works and measurements shall be carried out under industrial conditions. The present work scope should include an analysis of geometrical and technological process parameters. This enables the working space of the metallurgical reactor and the physicochemical properties of the media being modelled to be rendered precisely. The importance of this step is demonstrated by the fact that most of the time is dedicated thereto. It is estimated that it takes up to 70% of the time dedicated to the entire task solution process. It includes geometric model mathematical definition, selection of discretisation point locations and computational mesh generation, mathematical formulation of boundary and initial conditions, mixture physicochemical properties, or complementary model selection.

### 2.1. Numerical Modelling

Within the preparatory work frame, the computational mesh was tested and the turbulence model and initial and boundary conditions selection were performed. The numerical model adopted for numerical calculations is in accordance with results obtained during test calculations.

Quantitative analysis showed very slight differences in the liquid steel velocity inside the ladle between variants A (466,233 computing cells) and B (880,869 computing cells). Therefore, mesh containing 466,233 volumes was assumed to be dense enough to be used for main CFD calculations. It is also worth mentioning that the increase in calculation time between variants A and B is 96%.

Additionally, the quality of computational meshes was checked using the angle of skew criterion [15]. Normalizing in the range <0–1> value of the Q_EAS_ factor did not exceed 0.75 [15], which identifies the generated computational mesh skew angle allowed.

In the case of turbulent flow, determining liquid motion parameters in the near-wall layer requires to use of a very dense computational mesh to disclose characteristic distribution in the viscous sublayer. This involves significant calculation extension, therefore, in many engineering applications, so-called “wall function” SWF (Standard Wall Functions) is utilized, which within this range utilizes analytical solutions. Wall function using requires to occur at a distance of first computational mesh node from the wall in the logarithmic distribution zone. This distance can be controlled and determined based on y+ parameter, which should be within the range of 30 ÷ 60 [15]. During calculations, the y+ parameter was 43.

The potential of modern mathematical modelling techniques and CFD (Computational Fluid Dynamics) simulations enable liquid steel structure in the steelmaking ladle and, consequently, determining the time needed to achieve the required homogenisation state to be understood. Numerical simulations of the liquid steel purging with Argon were carried out using ANSYS Fluent [15] commercial code.

The following assumptions were adopted to model the steel mixing process in the ladle:

- Liquid steel flow is turbulent.
- Argon injection for forced convection makes flow two-phase one.
- Neglecting the presence of the slag-protecting metal—the metal surface was modelled as the flat free surface.
- Liquid steel’s natural convection influence on the mixing process is negligible.
- Alloy additive addition to liquid steel bath results in multi-component flow.
- Flow parameters associated with gas injection and alloy additive (tracer) addition are changing over time resulting in unstable liquid flow.
- Argon is injected through a porous plug of slotted type (number of slots—12) and innovative module at a defined mass flow rate (Q_m_), generating bubbles of a defined diameter.

The CFD numerical simulations are carried out with the commercial code Fluent, based on the Reynolds Averaged Navier Stokes (RANS) equations. The liquid phase Navier-Stokes equations for incompressible flow without source terms are as follows:

Continuity equation:

(1) $$\frac{\partial u_{i}}{\partial x_{i}}=0$$

Momentum conservation equation:

(2) $$\rho \frac{\partial u_{i}}{\partial t}+\rho u_{j}\frac{\partial u_{i}}{\partial x_{j}}=-\frac{\partial p}{\partial x_{i}}+\frac{\partial }{\partial x_{j}}\left[\mu_{eff}\left(\frac{\partial u_{i}}{\partial x_{j}}+\frac{\partial u_{j}}{\partial x_{i}}\right)\right]+\rho g_{i}$$

where

(3) $$\mu_{eff}=\mu +\mu_{t}$$

*u_i,j_* is time-averaged fluid velocities in the *i*th and *j*th directions respectively, *ρ* is liquid density, *p* is pressure in the fluid, *µ* is molecular viscosity of liquid, *µ_t_* is turbulent viscosity, *µ_eff_* is effective turbulent viscosity, *g_i_* is gravitational acceleration in the *i*th direction, *x_i_* and *x_j_* are spatial coordinates in the *i*th and *j*th directions, respectively, *i* and are the three directions in the global Cartesian coordinate system.

The energy equation can be written as:

(4) $$\frac{\partial }{\partial t}\left(\rho E\right)+\frac{\partial }{\partial x_{i}}\left[u_{i}\left(\rho E+p\right)\right]=\frac{\partial }{\partial x_{j}}\left(k_{eff}\frac{\partial T}{\partial x_{j}}+u_{i}{\left(\tau_{ij}\right)}_{eff}\right)$$

where *E* is the total energy, *k_eff_* is the effective thermal conductivity, and (*τ_ij_*)*_eff_* is the deviatoric stress tensor.

The realizable k-ε model, based on the Boussinesq approximation, indicating that the Reynolds stresses are a function of the mean gradients of velocity similar to the molecular stress, is used for modelling the turbulence.

The liquid phase is described by the equations in the Euler formulation, and the discrete phase (particles) is described by Lagrangian coordinates. For the calculations of the flow field the realizable k-ε turbulence model is used. The particle movement is simulated using the Discrete Phase Model (DPM). The differential equation describing the motion of particles in the liquid is [15]:

(5) $$\frac{du_{p}}{dt}=\frac{3\mu C_{D}{Re}_{p}}{4d_{p}}\left(u_{l}-u_{p}\right)+\frac{g(\rho_{p}-\rho_{l})}{\rho_{p}}+\frac{1}{2}\frac{\rho_{l}}{\rho_{p}}\frac{d}{dt}\left(u_{l}-u_{p}\right)+\frac{\rho_{l}}{\rho_{p}}u_{p}\frac{\partial u_{l}}{\partial x}$$

where: *u_p_* and *u_l_* are particle and liquid velocities, respectively, *ρ_p_* and *ρ_l_* are particle and liquid density, respectively, *d_p_* is particle diameter, *C_D_* is drag coefficient, *R_e_* is Reynolds number, *g* is gravitational constant, and *μ* is molecular viscosity.

During the research, unidirectional influence of bubble gas phase impacting the liquid steel was considered. However, in addition, stochastic component of gas bubble motion (Discrete Random Walk model, which was mentioned in this paper) was also considered, having on-purpose disturbance introduction into gas bubble motion [15]. In simulations considered, it was assumed that temperature value of Argon injected into the ladle was 300 K. At the current research stage, simplification in the form of constant gas bubble diameter with no heat transfer between continuous and discrete phases, which affect gas phase volume change, were introduced.

At the final stage of the calculations, variable approximation procedure is set to a second-order upwind. Discretisation equations were derived from the governing equations and solved using implicit finite difference procedure called SIMPLEC algorithm [15].

**Figure 3:**
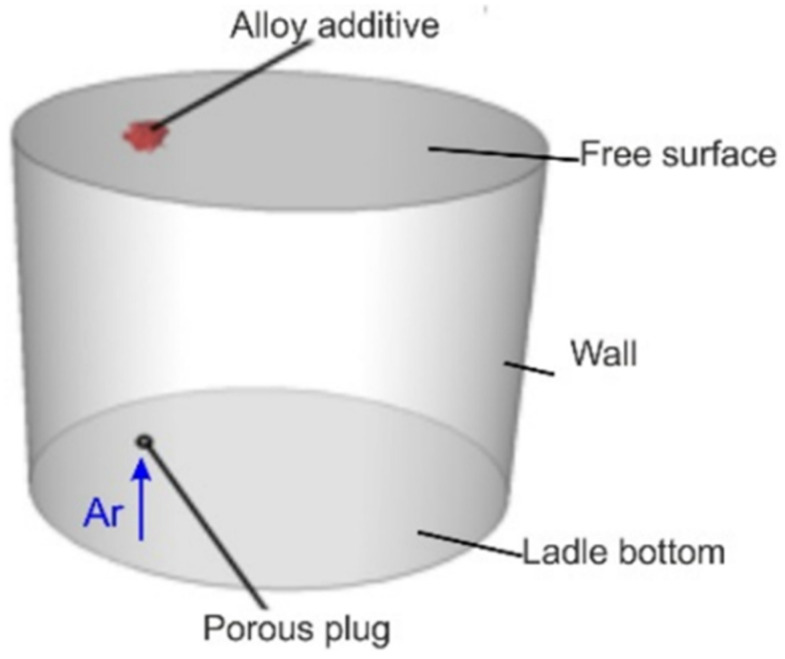
Ladle schematic diagram with boundary conditions applied.

The ladle schematic diagram, including the boundary conditions used for the numerical model, is presented in Figure 3.

CFD calculations were related to real industrial conditions. Therefore, real gas flow rates and geometrical parameters of the porous plug of slotted type and solution proposed—the gas-permeable module—were used for calculations. The module was installed centrally in the gas-permeable plug centerline.

The physicochemical properties of fluids used for CFD calculations are presented in Table 2.

Material data concerning the liquid steel for numerical simulations were assumed to be the same for both cases considered (existing technology and new solution proposed), which allow for a direct research result comparison. The data utilized for CFD calculations are provided in Table 3.

CFD simulations performed to determine mixing curves (Residence Time Distribution characteristics, RTD) were carried out for unsteady conditions, with iteration time step equal 0.5 s. In CFD calculations, it was assumed that the tracer introduction is given in a one portion. Alloy addition, 100 kg, is introduced in a strictly defined location—under metal bath surface, in centreline of the gas-permeable plug. Tracer has been added at fully developed flow (achieved flow structure in the ladle), exactly as it is in the real process.

The location of monitoring points is crucial for determining whether the steel-tracer mixture is already homogeneous. In a real process, only one monitoring point determines whether the mixture is homogeneous. To decrease the effects of CFD simulation data misinterpretation, it was decided to distinguish 15 monitoring points for the tracer concentration in the ladle working space. The monitoring points are located both—in zones with strong metal circulation (including directly in the gas-liquid column zone)—in zones with much lower liquid flow and mixing intensity (so-called ‘dead’ zones). Such monitoring point location variety ensures obtaining mixing characteristics representative of the steelmaking ladle’s entire volume.

### 2.2. Physical Modelling

**Figure 4:**
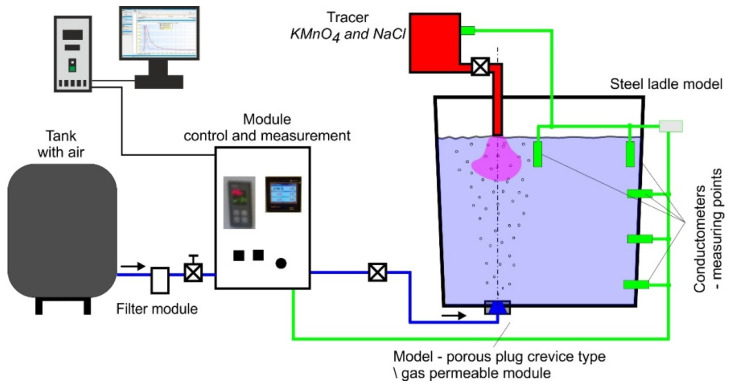
Test stand schematic diagram (steel ladle model).

Experimental laboratory investigations were carried out on the test stand (physical model) equipped with the ladle water model (WM) with the possibility of model liquid being purged by gases. The model is made in a linear descending scale of S_L_ = 1:3.4 (scaled down in accordance with Table 1) and allows to perform tests with inert gas. It is created in accordance with similarity theory principles [17,18,19]. In the model and the actual device, the characteristic criterion number compliance principle was applied. Modified Froude number (Fr) criterion [19,20,21] was used as the dominant similarity criterion. Based on this, the dynamic similarity of airflow through the model using the scaling method was set [19]. This model is described in detail in the work [22]. The model is equipped with specialised control and measuring equipment. The model schematic diagram is shown in Figure 4.

The design parameters and location of the porous plug/module were analogous to those in the CFD calculations and prepared according to the dimensional scale of the physical model. Values adopted for model tests, determined in accordance with the relationships [17,22], are presented in Table 4.

In research using the water model, the tracer (NaCl and KMnO4) introduction point is located in the centreline of the porous plug. Laboratory tests using the Ladle water model were divided into two stages, the first of which (qualitative) was aimed at determining the formation of the gas column and assessing the degree of gas dispersion in the volume of the model liquid. These studies were performed as visualisations. Their course consisted of recording the experiment in various planes using a camera system, computer processing of the recorded material, and finally, its interpretation. The second stage of the research (quantitative) was carried out to determine the effectiveness of the homogenization process of the model liquid under the influence of the gas blowing. It consisted of determining the minimum mixing time after the tracer introduction to the liquid bath, based on the analysis of the obtained mixing curve (RTD). To identify the mixing curve (quantitative research), the pulsed signal extortion method was utilized (Dirac’s method). When reaching assumed conditions (volume of gas being purged) and flow stabilisation in accordance with developed similarity conditions, a small tracer amount (NaCl aqueous solution) was provided into the system at one time.

Changes in model liquid electrical conductivity, representing changes in tracer (NaCl) concentration, were recorded continuously. Five conductivity meters were used to measure changes in model liquid conductivity, which were installed in selected points in the model workspace. Voltage generated at half-second time intervals by the conductivity meters is equivalent to changes in the tracer concentration in water. Signals from the conductivity meters registered by the recorder are further processed to plot the mixing curves.

## 3. Results and Discussions

### 3.1. Water Model Results

**Figure 5:**
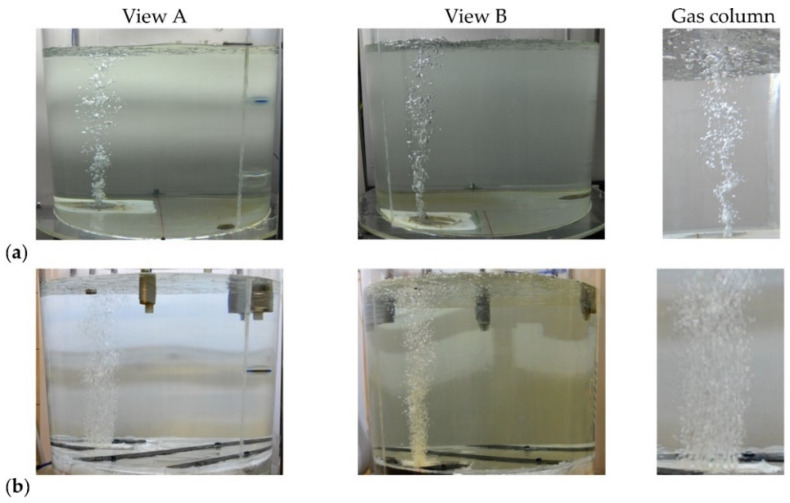
Gas bubble cone (gas column) formation mechanism and dispersion degree thereof in the model liquid (**a**) porous plug (of slotted shape) and (**b**) solution proposed, gas-permeable module.

During the research of visualisation nature, the experiment course was recorded using cameras placed in two measurement planes. Such camera arrangement enabled multi-planar model liquid circulation to be observed. Conducted research allowed the mixing process, gas bubble formation (gas column), dispersion thereof in the model liquid volume, and the gas column formation to be fully identified. Different experiment variants analysed are shown in Figure 5.

The photos in Figure 5 show a clear difference in the gas column structure. In the case of standard gas-permeable plug, the gas-liquid column is compact at the plug base, whereas as rising towards the surface, it becomes less homogeneous. The rising gas bubbles combine themselves into larger clusters, forming as it were, ribbons that move oscillating irregularly. In case of innovative gas-permeable module, the gas-liquid column is more dispersed and homogenous over the entire height. This phenomenon is very advantageous for liquid steel refining and mixing process, proceeding after alloy additive introduction.

Another issue undertaken as research part was tracer (KMnO_4_) dispersion analysis in the steelmaking ladle model. This is analogue of alloy addition dispersion in metal bath in the steelmaking ladle, at the ladle furnace station. This research method allows mixing process to be visualized and dead zones created in the ladle working space during this process can be located. Results obtained at this research stage allow research being carried out to be preliminary assessed.

**Figure 6:**
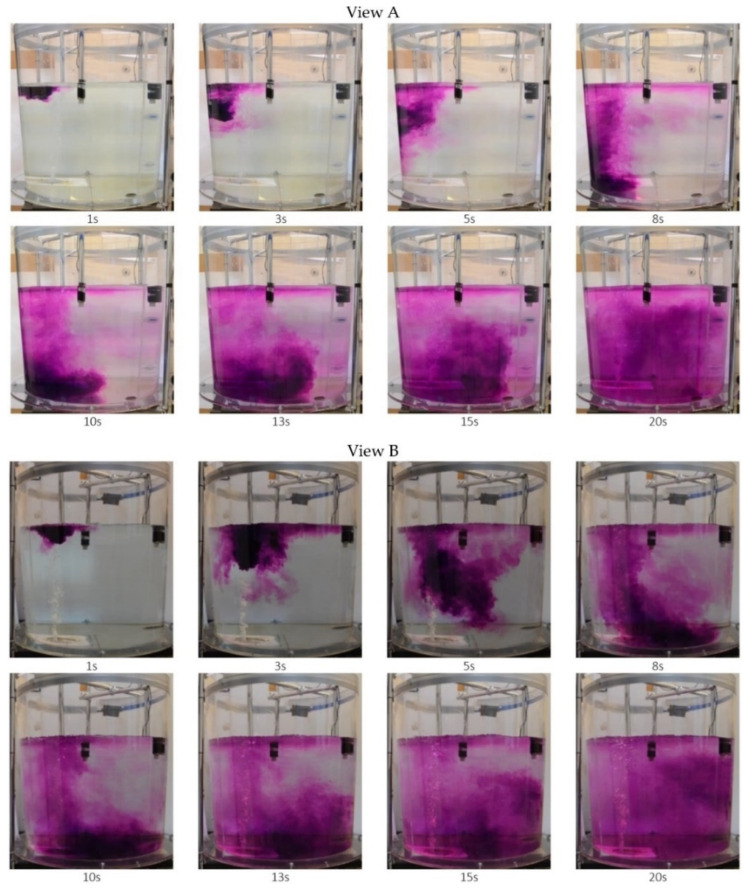
Test results illustrating model liquid mixing in the model steelmaking ladle, variant of experiment—the standard porous plug (slotted plug).

**Figure 7:**
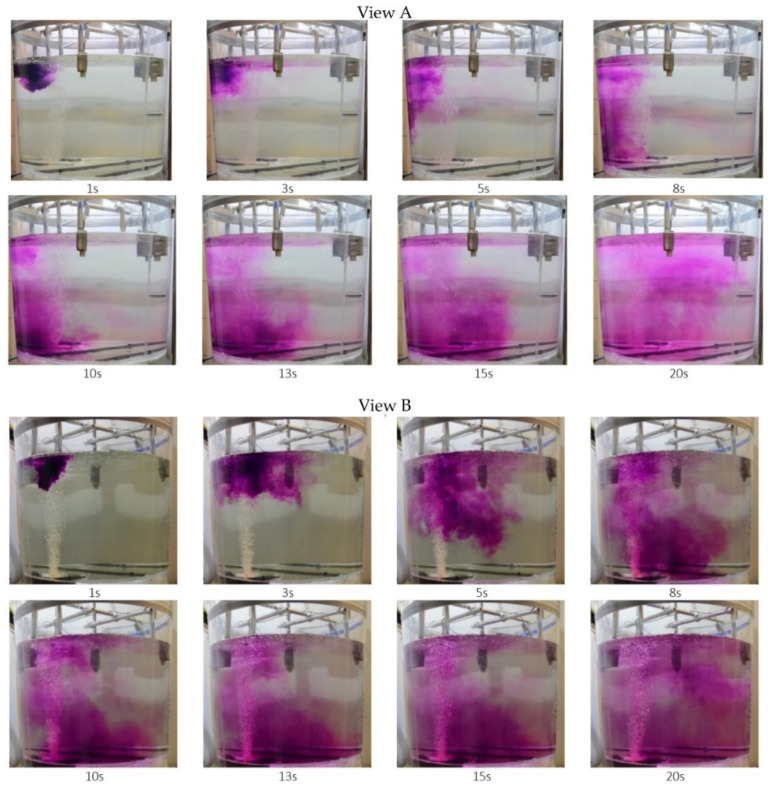
Test results illustrating model liquid mixing in the model steelmaking ladle, variant of experiment—modified module.

Example results of tracer flow dispersion are shown in Figure 6 and Figure 7. Visualization results are presented at two planes: view A from model front and view B from model side.

From analysis of photos presented in Figure 6 and Figure 7 clear differences in tracer dispersion process can be seen. This is due to changes in the liquid flow structure in the ladle, caused by gas introduction through the proposed innovative module. In the case of standard plug (see Figure 6), the tracer—in more concentrated form (higher concentration)—moves towards the ladle bottom, after which it is moved by rising stream and dispersed throughout the entire ladle volume. Whereas, in case of gas injected through the innovative module (see Figure 7), the tracer almost immediately becomes dispersed (after approximately 5 s). It is also directed towards the ladle bottom and lifted by rising liquid stream; however dispersion degree thereof is much higher.

### 3.2. CFD Simulation Results

**Figure 8:**
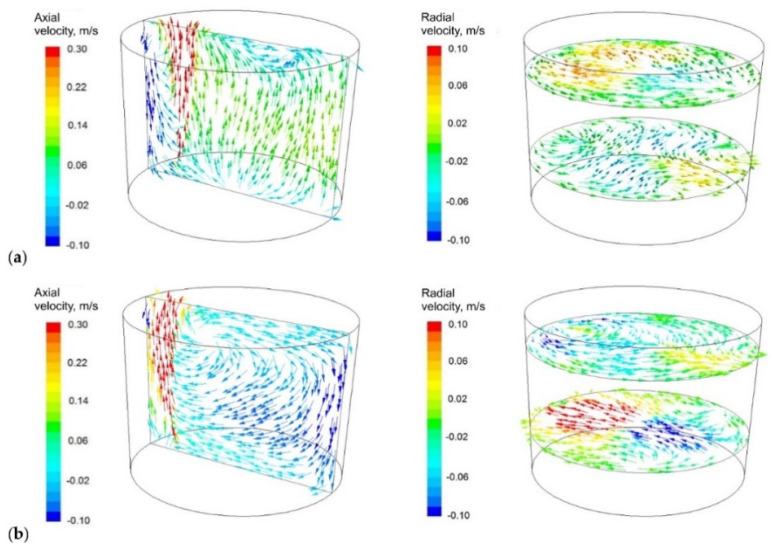
Velocity vector distribution onto the characteristic steelmaking ladle planes (**a**) current technology and (**b**) solution proposed.

**Figure 9:**
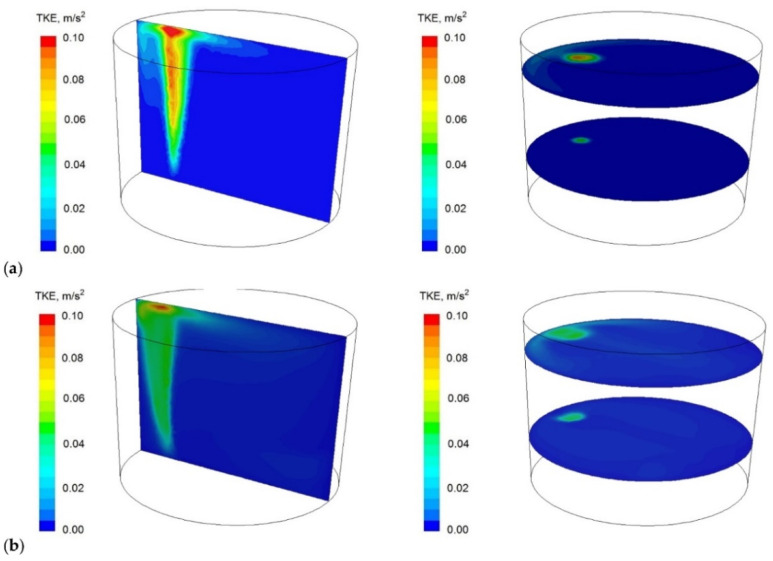
Turbulence kinetic energy (TKE) contour maps onto the steelmaking ladle planes selected (**a**) current technology and (**b**) solution proposed.

**Figure 10:**
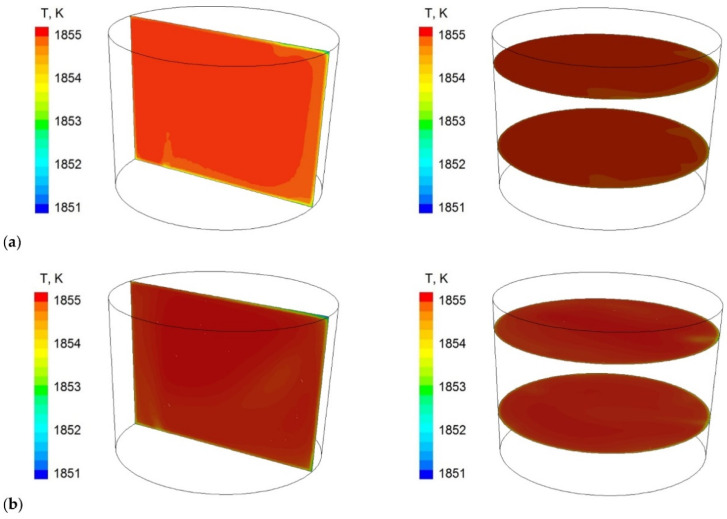
Contour maps of liquid steel temperature at selected planes of the steel ladle (**a**) current technology and (**b**) proposed solution.

Figure 8, Figure 9 and Figure 10 predicted the results of the values calculated concerning the technology currently used; proposed module are shown.

Figure 8 shows the distribution of velocity vectors on the characteristic steelmaking ladle planes for existing technology and the proposed module (the gas-permeable module). Very clear changes in the liquid flow structure in the ladle which have a strong influence on the previously observed changes in the tracer dispersion (Figure 6 and Figure 7), recorded during laboratory experiments on the water model, can be seen. 

Module using in the ladle workspace significantly changed circulation pattern. Two distinctive circulation areas (vortices) that dominated the flow, in contrast to the standard gas-permeable plug, for which this flow is less regular, can be clearly seen. This also affects turbulence kinetic energy distribution in the ladle, Figure 9 (it covers larger areas in case of the module), and metal bath thermal homogenisation, Figure 10 (more uniform in case of module using).

### 3.3. CFD Simulation and Physical Modelling Result Comparison to Verify Suitability of New Solution Proposed

Important issue is selection of process parameter that will allow for the technology currently used and solution proposed, the gas-permeable module to be compared. Such parameter shall be selected, which objectively allows to be determined and, at the same time, it is important in terms of research objective. Therefore, the most appropriate criterion for quantitative comparison will be comparison of alloy additive mixing time obtained for both solutions.

Metal bath chemical composition homogenisation is fundamental task of the ladle processes. This takes place throughout whole process, but it is still valuable to know the time needed to achieve desired bath chemical homogenisation degree after alloy addition introduction. The alloy addition dispersing process in the ladle is based on convection, molecular, and turbulent diffusion principles. From practical point of view, alloy addition dispersing in the metal bath is considered as macromixing process [23,24,25]. Moment, which is defined by following relationships, of reaching sufficient degree of bath homogenisation *Y* is considered as effective completion of such process:

(6) $$Y=C_{b}\cdot 100\%$$

(7) $$C_{b}=(C_{t}-C_{0})/(C_{\infty }-C_{0})$$

where *C_t_*, *C_0_*, and *C*_∞_ are tracer concentrations at time *t* at the beginning and at the end of the process, respectively.

Theoretically, it is assumed that mixing is fulfilled when bath homogenisation degree achieves 95%, which is corresponding to *C_b_* ∈ 〈0.95;1.05〉, or fulfilled, when bath homogenisation degree achieves a level of 99.5%, which is corresponding to *C_b_* ∈ 〈0.995;1.005〉. Ranges shown in parentheses indicate dimensionless range of the tracer concentrations (component tested in the steel) [17,23]. At in-house analysis, 95% bath homogenisation degree was assumed in view of fact that it is commonly used under standard conditions in majority of steel plants.

**Figure 11:**
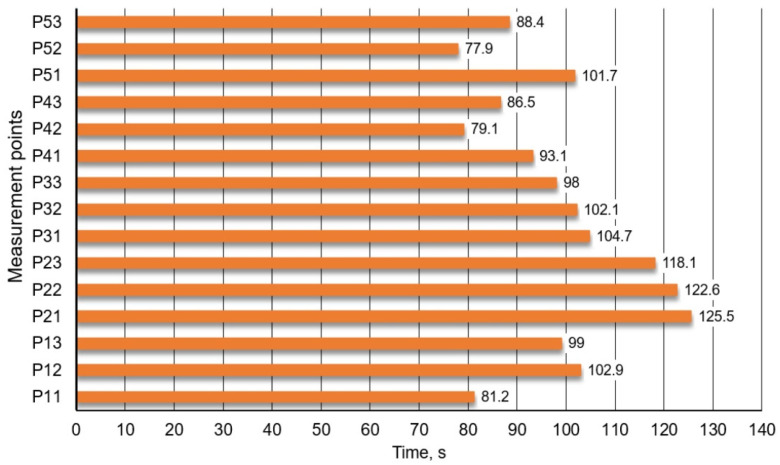
Exemplary specification (for CFD) for mixing time (at individual monitoring points), variant of technology currently used, porous plug (slotted type).

In Figure 11 mixing times obtained for 15 specific monitoring points are given. Results concerning the currently used technology variant, porous plug of slotted type, for which 95% homogenization degree was achieved.

**Figure 12:**
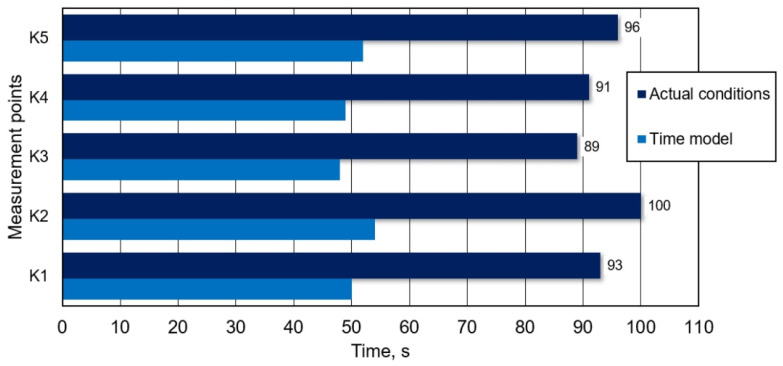
Exemplary specification (water model) for mixing time at individual monitoring points, variant, the gas-permeable module proposed.

Figure 12, in turns, shows mixing times obtained for 5 individual monitoring points, for proposed innovative solution, gas-permeable module, for which 95% homogenization degree was achieved.

It follows from data (Figure 11 and Figure 12) that determined mixing time depends on monitoring point location (in zone of so-called stagnant flows or dead zone, it is significantly longer); therefore, it was decided that longest mixing time thereof shall be treated as total mixing time.

To directly compare results obtained from CFD calculations with results of experiments carried out on the ladle physical model, they were converted into real conditions with using time scale, which depends on the linear scale in accordance with relationship:

(8) $$S_{t}=\sqrt{S_{L}}$$

**Figure 13:**
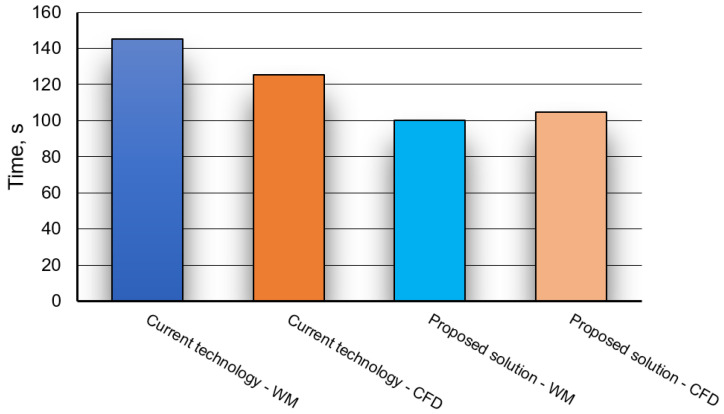
Quantitative comparison of alloy additive mixing times obtained for both solutions (technology currently used and solution proposed, the gas-permeable module).

Based on model tests carried out (physical modelling and CFD) and performing suitable converting calculations in accordance with relationship (3) for the physical model, results of alloy additive mixing time obtained for both methods are presented in Figure 13.

Additionally, the relative error was calculated for both methods. It is defined according to the relationship:

(9) $$\delta_{i}=\frac{t_{m}^{WM}-t_{m}^{CFD}}{t_{m}^{WM}}\cdot 100\%$$

The calculated relative error values are presented in Table 5.

Table 5 shows that the error value for the current technology is much higher than for the proposed solution. The error value between the results obtained from the experiment and CFD calculations for both solutions does not exceed 13.4%. Therefore, it can be concluded that satisfactory compliance of the CFD results with the results obtained using the water model was obtained. This statement is justified by the fact that an error of 10–20% is generally considered acceptable when assessing reliable (industrial) flow characteristics for large-sized facilities. This error is also at an acceptable level for the industrial side, which allows further tests to be carried out on the industrial facility.

Noticeable differences between results for water model and CFD, current technology and solution proposed, can be justified by the following factors:

- harder to catch starting moment in water model,
- precision of tracer insertion in water model,
- numerical idealisation of tracer introduction and/or simplifications used in the CFD model.

The obvious answer will come from industrial tests carried out in a steel plant. It should be mentioned here that reducing the mixing time by even a few minutes for the technological process brings measurable benefits, even financial ones.

However, analysing the results presented in Figure 13 (taking into account the error) in terms of the technological process carried out in ladle, it follows that the gas-permeable module clearly influences alloy addition mixing time. This may be particularly important for industrial steelmaking conditions. In practice, there is often a necessity to accelerate the metal processing process to ensure continuity of continuous steel casting process. 

By using an innovative module, process time reduction is significant, which was confirmed both by physical (water) model tests and CFD simulations. Considering an error of about 20% (model tests), this solution is still acceptable for the industry partner and introduces sufficient innovation in a secondary metallurgy technology.

**Figure 14:**
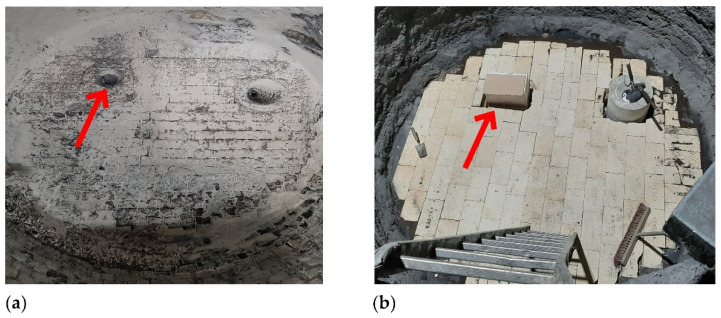
View of (**a**) the porous plug installed in the steelmaking ladle bottom and (**b**) the solution proposed- the gas-permeable modified module.

Based on hybrid research results, the innovative module was developed which will be used to carry out industrial research at project’s next stage. Figure 14 shows a view of a traditional plug fixed in the steelmaking ladle bottom location and the solution proposed, the gas-permeable module.

## 4. Conclusions

In high-quality steels, the quantity of non-metallic inclusions, and chemical and structural homogenisation high degree are very important. Refining processes occur in the secondary metallurgy stations. The ladle furnace is one of the aggregate units in which appropriate conditions for refining and for achieving chemical and thermal homogenisation prevail. By purging inert gas into the bath, a desirable liquid steel flow structure in the ladle can be achieved. In this case, it is essential not only gas flow rate but also gas-permeable plug (plugs) location in the ladle and construction itself thereof.

Introducing new technological solutions into industrial practice, having on purpose to improve production and economic results achieved so far, requires conducting numerous preceding research.

Model research was carried out to test and compare the innovative gas-permeable module with the plug currently used in the steelmaking ladle for technological conditions of Cognor SA Ferrostal Łabędy Steelworks in Gliwice. To carry out research, a hybrid modelling approach (mathematical modelling and physical modelling) was used. The research revealed the benefits of the gas-permeable module proposed. It was found that:

- The use of a newly proposed gas-permeable module changes the previously obtained structure of the gas-liquid column. The use of the module causes the formation of a large number of small gas bubbles, which helps to increase the refining capacity.
- Due to the strong fragmentation of the gas bubbles, the growth of the gas column at the metal surface is small, which has a beneficial effect on the free surface waving phenomenon.
- For the same reason, the interaction of the gas column with the refractory lining is also weakened. This limits the risk of secondary contamination of the metal bath.
- Increasing the active surface of the module has a beneficial effect on increasing the degree of gas dispersion in the metal bath and, consequently, improving the refining capacity.
- Verification (using the water model) of adopted assumptions and calculation procedures necessary for the description of liquid movement and mixing in the ladle tested, indicates data good quality compliance.
- Module using significantly changes the circulation pattern in the ladle workspace. There occur two distinct circulation areas (vortices) that dominate the flow. It also affects turbulent kinetic energy distribution in the ladle (it covers larger areas) and has a direct effect on the metal bath thermal homogenisation degree.
- The new gas-permeable module using causes the gas-liquid column to be more dispersed and homogenous along the entire height. This brings a positive effect on the liquid steel mixing process after alloy addition introduction.
- The liquid flow structure in the ladle caused by gas introduction through the module proposed results in much faster tracer (alloy additive) dispersion in object volume as compared with the process course with the standard tracer using. Thus, the gas-permeable module using has a clear effect on alloy additive mixing time reduction as compared with the plugs currently used.

## Figures and Tables

**Table 1:** Ladle construction parameters.

Parameter	Symbol	Unit	Value
Diameter	A	m	2.456
	A1		2.616
Radius	R		1.228
	R1		1.308
Ladle height	H		2.322
Ladle height (liquid steel level)	h		1.840
Position of the fitting	L_K1_		0.500
	L_K2_		0.600

**Table 2:** Properties of liquids used for CFD calculations.

Fluid	Property	Value	Unit
Liquid steel	Temperature	1858	K
	Density	7011	kg·m^−3^
	Viscosity	6.7	kg·m^−1^·s^−1^
Argon	Density	1.634	kg·m^−3^

**Table 3:** Data used for CFD calculations.

Steel Grade	Liquid Steel Temperature in Ladle, °C	Liquid Steel Density, kg·m^−3^	Amounts of Alloy Addition, kg	Gas (Ar) Intensity, dm^3^·min^−1^	Heat Flux Losses [16], kW·m^−2^ by	
					Surface	Walls and Bottom
B500B	1585	7011	100	200	12.5	5.0

**Table 4:** Data set for research using the water model.

Gas (Ar) Intensity—Industry dm^3^·min^−1^	Gas (O_2_) Intensity—Model dm^3^·min^−1^	Water Density kg·m^−3^	Tracer	
			Quantitative Research—Mixing Curves	Qualitative Research—Visualization
200	3.7	997	NaCl	KMnO_4_

**Table 5:** Relative error of the mixing time determined in CFD simulations to that determined experimentally WM.

Variant	Error Value (%)
Current technology—porous plug (slotted type)	13.4
Solution proposed—innovative module	−4.5

## Data Availability

Data are contained within the article.
